# Selecting short length nucleic acids localized in exosomes improves plasma *EGFR* mutation detection in NSCLC patients

**DOI:** 10.1186/s12935-019-0978-8

**Published:** 2019-10-01

**Authors:** Yoonjung Kim, Saeam Shin, Boyeon Kim, Kyung-A Lee

**Affiliations:** Department of Laboratory Medicine, Yonsei University College of Medicine, Seoul, Republic of Korea

**Keywords:** Liquid biopsy, Extracellular vesicles, Circulating tumor DNA, Non-small cell lung cancer, Epidermal growth factor receptor, ddPCR

## Abstract

**Background:**

Exosomal nucleic acid (exoNA) is a feasible target to improve the sensitivity of *EGFR* mutation testing in non-small cell lung cancer patients with limited cell-free DNA (cfDNA) mutant copies. However, the type and size of target exoNA related to the sensitivity of *EGFR* mutation testing has not been explored extensively.

**Methods:**

The type and size of target exoNA related to the sensitivity of *EGFR* mutation testing was evaluated using ddPCR. A total of 47 plasma samples was tested using short-length exoTNA (exosomal DNA and RNA) and cfDNA.

**Results:**

The sensitivity of short-length exoTNA (76.5%) was higher than that of cfDNA (64.7%) for detecting *EGFR* mutations in NSCLC patients. In *EGFR*-mutant NSCLC patients with intrathoracic disease (M0/M1a) or cases with low-copy T790M, the positive rate was 63.6% (*N* = 7/11) and 45.5% (*N* = 5/11) for short-length exoTNA and cfDNA, respectively. On average, the number absolute mutant copies of short-length exoTNA were 1.5 times higher than that of cfDNA. The mutant allele copies (Ex19del and T790M) in short-length exoTNA were relatively well preserved at 4 weeks after storage. The difference (%) in absolute mutant allele copies (Ex19del) between 0 days and 4 weeks after storage was − 61.0% for cfDNA.

**Conclusion:**

Target nucleic acids and their size distribution may be critical considerations for selecting an extraction method and a detection assay. A short-length exoTNA (200 bp) contained more detectable tumor-derived nucleic acids than exoDNA (~ 200 bp length or a full-length) or cfDNA. Therefore, a short-length exoTNA as a sensitive biomarker might be useful to detect *EGFR* mutants for NSCLC patients with low copy number of the mutation target.

## Background

The identification of driver and resistance mutations located in the tyrosine kinase domain of *EGFR* in a subset of non-small cell lung cancer (NSCLC) is necessary to guide patient treatment options and to predict prognosis [1–3]. The majority of *EGFR* genotyping is assessed through conventional tumor biopsy. However, this process can frequently put the patient at risk and may miss portions of the tumor that are developing treatment resistance or have acquired new driver mutations due to tumor heterogeneity [4]. Thus, liquid biopsy is considered an alternative for detecting resistant-*EGFR* mutants in NSCLC patients undergoing tyrosine kinase inhibitor (TKI) therapy [5]. Circulating tumor DNA (ctDNA) is the most common source of nucleic acid for detecting *EGFR* and is already being implemented in clinical practice [6]. Current technologies, such as polymerase chain reaction (PCR)-based method and next-generation sequencing, have been developed to detect very low level of mutant ctDNA fraction in plasma [7, 8]. However, these platforms show varying sensitivity depending on mutations and are not satisfactory enough to get reliable results in patients with scant T790M copies (< 0.01%) [9–11]. Since tumor-derived nucleic acids rarely exist existing in patient’s plasma, pre-analytical steps, especially selecting target materials that harbored relatively abundant mutant alleles and extracted them using an appropriated method, are important to improve the sensitivity of *EGFR* test using patient’s plasma with low levels of mutant alleles.

Exosomes are endosome-derived small membrane-bound vesicles that are released by different cell types [12]. Exosomes carry proteins, lipids, and nucleic acids, including DNAs and RNAs [13]. Growing evidence have indicated that exosomes are associated with tumor development and metastasis [13, 14]. Exosomal nucleic acids (exoNA) containing tumor-derived nucleic acids were studied as a target for cancer mutation testing, and they also emerged as biomarkers for cancer diagnosis [15]. Moreover, they have recently been reported to be a sensitive source for *EGFR* genotyping [16, 17]. To improve the sensitivity of detection in patients with limited mutant copies of cfDNA, such as those with early-stage NSCLC or intrathoracic disease (M0/M1a), exosomal nucleic acid (exoNA) might be a feasible alternative [15, 18]. To acquire high quality and quantity of exoNAs, the type and size of target exoNA should be considered when choosing an extraction method; however, the type and size of target exoNA related to the sensitivity of *EGFR* mutation testing have not been extensively studied. In previous studies, the target of exoNA was exoDNA, exoRNA, or combined exoDNA/RNA [15–17, 19, 20]. Some studies targeted high molecular DNA from exosomes [17, 21], and other studies focused on fragmented DNA that localized in exosomes [22]. In this study, we evaluated exoNAs to carefully determine a sensitive circulating biomarker in a plasma *EGFR* genotyping assay. Our results demonstrate that short-length exoTNA (exosomal DNA and RNA) is a feasible target in patients with low-level *EGFR* mutant copies. We extracted short-length exoTNA using specific extraction kits that could enrich tumor-derived ~ 200-bp-sized NAs. We then compared the ExoNAs and cfDNA isolated from NSCLC patients using droplet digital PCR (ddPCR) to detect *EGFR* mutations including exon 19 deletion (Ex19del), T790M, and L858R.

## Materials and methods

### Study design

ddPCR assays were performed with the PrimePCR™ ddPCR™ Mutation Detection Assay kit and PrimePCR™ ddPCR™ *EGFR* Exon 19 Deletions Screening Kit (Bio-Rad Laboratories, Hercules, CA, USA) (Additional file 1: Table S1). We selected three hotspot mutations of Ex19del, L858R, and T790M. L858R and Ex19del are the most common forms of *EGFR* sensitizing mutations (85%) that are responsive for EGFR TKI treatment [23]. In case of progression on 1st generation TKI treatment, T790M mutation testing is recommended as acquired T790M mutation is the most common resistance mechanism (> 50%) that is responsive for 3rd generation TKI treatment [23, 24]. The limitation of detection (LOD) was determined as the lowest mutant concentration above the 95% confidence interval (CI) of the wild-type (WT) control, which was determined using a Poisson model (Additional file 1: Table S2). Validation ddPCR was performed using Multiplex I cfDNA Reference Standard (Horizon Discovery, Cambridge, UK) (Additional file 1: Table S3). The ability to detect *EGFR* mutation based on type of input nucleic acid (short-length exoTNA and a size range of exoDNA) was evaluated (Additional file 1: Figures S1 and S2). Analytic performance of isolated cfDNA and short-length exoTNA was evaluated using ddPCR. We assessed the influence on cfDNA levels and short-length exoTNA according to storage period.

### Patients

From November 2017 to November 2018, 47 NCSLC patients were consented and enrolled. *EGFR* genotyping results (*N* = 46) from the corresponding tissue specimens were obtained. Patients consented to the protocol approved by the Institutional Review Board of Gangnam Severance Hospital and Kangnam Sacred Heart Hospital. Plasma (2 mL) was collected from a total of 47 patients and stored at − 80 °C.

### Nucleic acid extraction and cDNA synthesis

We extracted plasma cfDNA using the MagMAX Cell-Free DNA Isolation Kit (Thermo Fisher Scientific, Waltham, MA, USA). Exosomes were isolated from plasma using ExoQuick™ (System Biosciences, Mountain View, CA, USA). Subsequently, short-length exoDNA and short-length exoTNA were isolated by MagMAX Cell-Free DNA Isolation Kit (Thermo Fisher Scientific) and MagMAX™ Total Nucleic Acid Isolation Kit (Thermo Fisher Scientific), respectively. A size range of exoDNA was isolated by the QIAamp DNA Blood Mini Kit (Qiagen, Hilden, Germany). The concentration and size distribution of cfDNA and exoNA were assessed using a 2200 TapeStation Instrument (Agilent Technologies, Santa Clara, CA, USA) with the Agilent High Sensitivity D1000 ScreenTape System and Genomic DNA ScreenTape System. The RNA yield and size distribution were analyzed using an Agilent 2100 Bioanalyzer with an RNA 6000 Pico kit (Agilent Technologies, Foster City, CA, USA). cDNA synthesis was performed using a SuperScript™ VILO™ cDNA Synthesis Kit (Invitrogen, Carlsbad, CA, USA).

### Validation of ddPCR

The ddPCR assays were performed with the PrimePCR™ ddPCR™ Mutation Detection Assay kit and PrimePCR™ ddPCR™ *EGFR* Exon 19 Deletions Screening Kit (Bio-Rad Laboratories) (Additional file 1: Table S1). We used cfDNA from Multiplex I cfDNA Reference Standards (Horizon Discovery) that included wild-type cfDNA with mutant allele frequencies of 5%, 1%, and 0.1%. cfDNA Reference Standards (Horizon Discovery) with 0.1% mutant allele was serially diluted to wild-type cfDNA for analytical sensitivity of the ddPCR assay (Additional file 1: Table S3). Healthy control samples and DNA-free samples were also analyzed (Additional file 1: Table S2) [25, 26]. Amplifications were carried out in a reaction volume of 20 μL on a QX100 Droplet Digital PCR System (Bio-Rad). The 20 μL PCR mix was composed of 10 μL Bio-Rad Super mix TaqMan, 1–2 μL of each amplification primer/probe mix, and 8–9 μL NAs. Thermal cycling comprised an initial denaturing and polymerase hot-start activating step of 10 min at 95 °C, followed by 40 cycles of 95 °C for 30 s and 55 °C for 60 s. Results were analyzed with QuantaSoft v.1.7.2 software (Bio-Rad) and reported as copies per milliliter of plasma.

### Effects of storage on cfDNA and short-length exoTNA concentrations

Samples from two patients with *EGFR* mutation and three normal controls were collected in K_2_ EDTA tubes. Immediately separated plasma was aliquoted into three tubes per sample and stored at − 80 °C. We assessed the influence of storage period on cfDNA levels and short-length exoTNA extracted at different time points (0, 14, and 28 days).

### Data analysis

Quantification of the number of target DNA molecules in the reaction is achieved by counting the number of positive and negative droplets. The LOD was determined as the lowest mutant concentration above the 95% CI of the WT control. The 95% CI was determined using a Poisson model and CLSI EP17-A2 [26, 27]. Details are described in Additional file 1: Table S2. Assays were considered “positive” if the measured event rate was ≥ 2 events/assay and “negative” if the event rate within a gated region was < 2 events/assay.

### Statistical analyses

Statistical analysis was performed using R (version 3.5.2, http://www.r-project.org) and MedCalc software (https://www.medcalc.org/). Data are presented using a 95% CI and 2-sided *P* value.

## Results

### Patient characteristics

The patients’ characteristics are described in Table 1. Patients had a median age of 73 years (range, 52–85 years), and 19 patients (40.4%) were female. The adenocarcinoma, squamous cell carcinoma, and other histologic types numbered 32 (68.1%), 9 (19.1%), and 6 (12.8%), respectively. Stage IV was dominant (*N* = 31, 66.0%), and other stages (I–III) represented 34.0% of total patients. Patients with intrathoracic metastatic disease (M0/M1a) accounted for 51.0% (Table 1).

**Table 1:** Baseline characteristics of patients

Characteristic	All patients (*N* = 47)^a^
Age (years)	73 (52–85)
Gender	
Female	19 (40.4%)
Male	28 (59.6%)
Histologic type	
Adenocarcinoma	32 (68.1%)
Squamous cell carcinoma	9 (19.1%)
Other	6 (12.8%)
Tumor stage	
I	5 (10.6%)
II	2 (4.3%)
III	9 (19.1%)
IV	31 (66.0%)
M category^b^	
M0	16 (34.0%)
M1a	8 (17.0%)
M1b	3 (6.4%)
M1c, single organ	3 (6.4%)
M1c, multi organs	17 (36.2%)
Chemotherapy	
TKI-naïve	43 (91.5%)
TKI-treated	4 (8.5%)

*Other* not otherwise specified, *TKI* tyrosine kinase inhibitor

^a^Results are expressed as median (range) or number (%)

^b^According to the 8th TMN edition, M1a indicates lung metastases or pleural/pericardial malignant effusion or nodules; M1b indicates a single metastatic lesion in a single distant organ; M1c indicates multiple lesions in a single organ or multiple lesions in multiple organs

### Assessment of ddPCR assay sensitivity

The analytical sensitivity of the ddPCR assay was evaluated using spiked samples with mutant allele frequencies of 1%, 0.1%, 0.02%, 0.01%, and 0.005% (Additional file 1: Table S3). The expected copy number of mutant alleles (1–32 copies) spiked into the wild-type alleles (2000–20,000) and the actual copy number of mutant alleles observed in the spiked samples are shown in Additional file 1: Table S3. An ultra-rare mutation (1 copy in a spiked sample) as low as 0.007–0.008% was successfully detected by the ddPCR assay. However, when we considered 2 copies/mL as a threshold for a positive result, Ex19del, L858R, and T790M were detected even at fractional abundance of 0.03%, 0.013%, and 0.018%, respectively (Additional file 1: Table S3). The limit of blank (LOB) defined by the frequency of positive droplets measured in DNA-free samples and the standard deviation (SD) of healthy controls were used to determine the lower LOD. Additional file 1: Table S2 shows the raw data for LOB analysis and LOD.

### Comparison between cfDNA and size-selectively extracted exoNAs

To elucidate the components of exoNAs that were related to the sensitivity of *EGFR* mutation testing, pooled plasma samples with Ex19del mutation were used. Isolated cfDNA, short-length exoNAs (DNA and TNA), and a full-length exoDNA including low and high molecular weight nucleic acids were co-isolated (Additional file 1: Figure S1). We assessed tumor-derived NAs to be more abundant in the short-length NAs (~ 200 bp long) than the full-length exoDNAs in exosomes (Additional file 1: Figures S1 and S2). Especially, short-length exoTNA is superior to other nucleic acid materials (cfDNA, short-length exoDNA, and full-length exoDNA) for detecting *EGFR* mutant alleles (Additional file 1: Figure S1). Quantity and size of short-length NAs were further confirmed by Bioanalyzer results, which also showed a major peak at less than ~ 200 bp long (data not shown).

### Mutant allele ratio between cfDNA and short-length exoNA

We evaluated the quantity of wild-type and mutant allele copies at cfDNA and short-length exoNAs (DNA and TNA) in spiked samples from NSCLC patients harboring *EGFR* mutation. The mutant allele ratio (short-length exoTNA/cfDNA) ranged from 1.2 to 2.5, and the mutant allele ratio of short-length exoDNA/cfDNA ranged from 0.0 to 1.0 (Table 2). Short-length exoTNA showed the largest absolute number of mutant allele copies compare to cfDNA and short-length exoDNA. However, due to the abundance of wild-type allele copies in short-length exoTNA, the mutant allele fraction (%) of cfDNA was generally higher than that of short-length exoTNA. Both cfDNA and short-length exoTNA are considered good materials for detection of tumor-derived mutant alleles (Table 2).

**Table 2:** Comparison between cfDNA and short-length exosomal nucleic acids

Sample^a^	Target	Mutant events	Wild-type events	Fraction (%)	Mutant allele ratio		Wild-type allele ratio	
					exoDNA/cfDNA	exoTNA/cfDNA	exoDNA/cfDNA	exoTNA/cfDNA
Spiked sample 1 (Ex19del)	cfDNA	20	418	4.78	0.5	1.2	0.6	2.7
	Short-length-exoDNA	9	270	3.33				
	Short-length-exoTNA	24	1138	2.11				
Spiked sample 2 (L858R)	cfDNA	1	156	0.64	1	2	0.8	2.6
	Short-length-exoDNA	1	131	0.76				
	Short-length-exoTNA	2	399	0.5				
Spiked sample 3 (L858R)	cfDNA	2	38	5.26	0	2.5	0	6
	Short-length-exoDNA	0	1	0				
	Short-length-exoTNA	5	228	2.19				
Spiked sample 3 (T790M)	cfDNA	0	97	0	N.A.	N.A.	N.A.	N.A.
	Short-length-exoDNA	6	89	6.74				
	Short-length-exoTNA	8	307	2.61				

*cfDNA* cell-free DNA, *exoDNA* exosomal DNA, *exoTNA* exosomal DNA and RNA, *Ex19del* exon 19 deletion, *N.A* not available

^a^Spiked samples with pooled plasma from patients harboring mutations in *EGFR* (Ex19del, L858R, and T790M). cfDNA and short-length exoDNA were extracted using MagMAX Cell-Free DNA Isolation Kit. Short-length exoTNA was extracted using MagMAX™ Total Nucleic Acid Isolation Kit

We performed ddPCR using 250, 500, 750, and 1000 μL plasma to assess the proper plasma volume for sensitive detection and monitoring. The amount of input plasma volume determined number of mutant allele copies in cfDNA and exoNAs. The mutant allele ratio between cfDNA and short-length exoTNA was relatively higher in a small input volume (250 μL) (Additional file 1: Figure S1).

### Quantification of short-length exoTNA and cfDNA in clinical samples

**Fig. 1:**
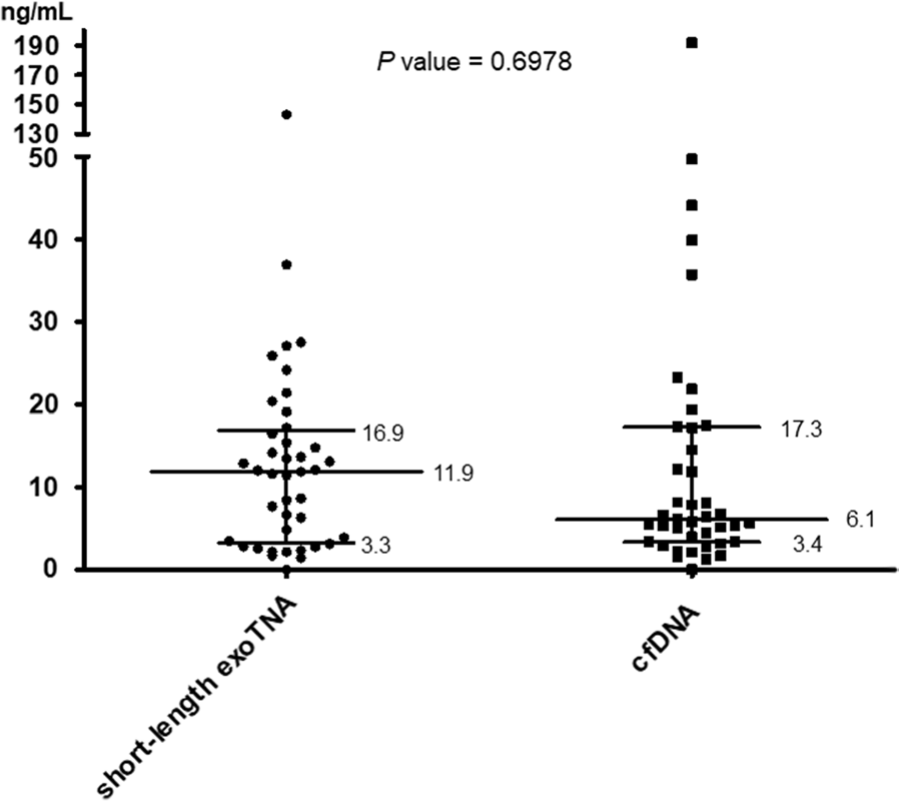
Nucleic acid concentration plots from short-length exoTNA and cfDNA. The y-axis indicates the concentration of nucleic acids in nanograms per milliliter, and the x-axis indicates sample materials. Black horizontal bars indicate median with interquartile range. The *P* value was calculated using paired t test

Short-length exoNA and cfDNA were extracted from equal volumes (1 mL) of plasma samples from 47 patients. Among them, 7 samples were not available for NA quantification due to lack of volume. The concentrations of short-length exoTNA and cfDNA are depicted in Fig. 1. The median concentration levels of short-length exoTNA and cfDNA were 11.9 ng/mL and 6.1 ng/mL, respectively, and there was not a significant difference between short-length exoTNA and cfDNA (*P* = 0.6978).

### Comparison of *EGFR* mutation status in NSCLC patients

**Fig. 2:**
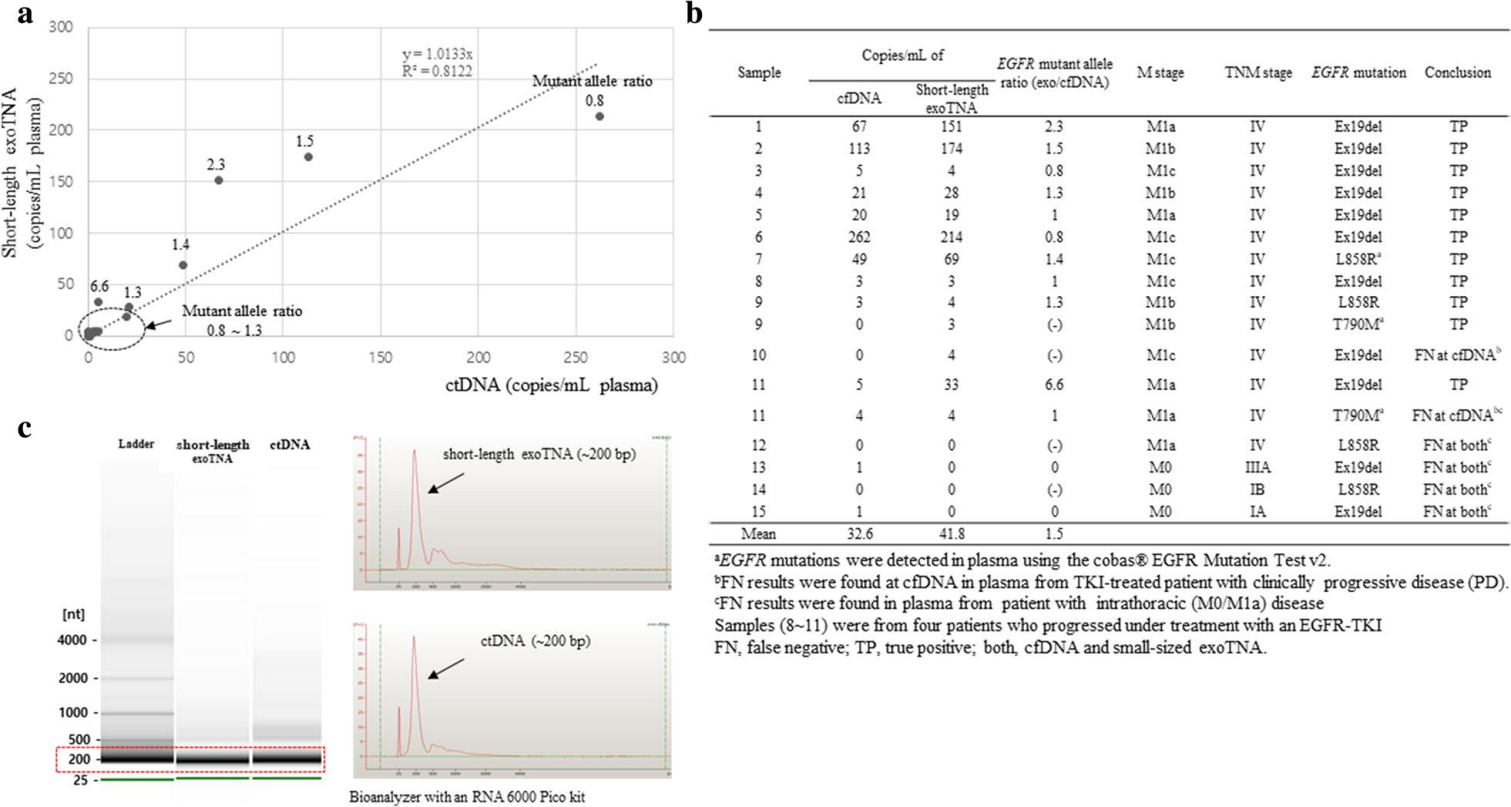
Comparison between short-length exoTNA and cfDNA in plasma from NSCLC clinical samples. **a**, **b** The mutant allele ratio (short-length exoTNA/cfDNA) and mutant allele copies/mL in NSCLC patients harboring *EGFR* mutation. **c** The size distribution of nucleic acids in cfDNA and short-length exoTNA in plasma from *EGFR* mutated NSCLC patients

Plasma samples from 47 patients were tested with short-length exoTNA and cfDNA using ddPCR, and each sample was analyzed for Ex19del, L858R, and T790M. Of the 141 measurements (3 mutant sites per each patient), 17 and 124 measures were classified as true positives (TP) and true negatives (TN), respectively, compared to tissue biopsy (Table 3). Seventeen TPs were detected from 15 patients; among them, 2 patients harbored both activating and resistant *EGFR* mutations (Fig. 2b). Three patients harboring T790M or L858R were only detected in plasma samples using the cobas^®^ *EGFR* Mutation Test v2 not in tissue samples. These *EGFR* mutations were also detected in cfDNA or/and short-length exoTNA up to a 9.5% mutant allele fraction (%). Therefore, we regarded these three mutations as “TP” results. The sensitivity of short-length exoTNA (76.5%) was higher than that of cfDNA (64.7%) for activating/resistant *EGFR* mutations (Table 3). We also found that 46.7% (7/15) of TP cases were from patients with intrathoracic (M0/M1a) disease, and 53.3% (8/15) were from patients with M1b/M1c disease. In false negative (FN) results, 9 FNs (short-length exoTNA, 4 samples; cfDNA, 5 samples) were from five patients with M0/M1a. One FN case (cfDNA, 1 sample) was from a patient who progressed under treatment with an EGFR-TKI (Fig. 2b). The specificity of *EGFR* genotyping was 100.0% and 97.6% for short-length exoTNA and cfDNA, respectively (Table 3). Two false-positives (FPs) were found only in cfDNA with two events of the mutant allele in ddPCR. The accuracy of *EGFR* genotyping was slightly higher with short-length exoTNA (97.2%) than with cfDNA (93.6%) (Table 3). The mean mutant allele ratio (short-length exoTNA / cfDNA) was 1.5 and ranged from 0.8 to 6.6 (Fig. 2a, b). The main peak of nucleic acids of cfDNA and short-length exoTNA was ~ 200 bp long in *EGFR* mutated NSCLC patient plasma (Fig. 2c).

**Table 3:** Comparison of the *EGFR* mutation status between tumor tissue and plasma in NSCLC patients (*N*=47)

*EGFR* genotype	TP and TN^a^	cfDNA		Short-length exoTNA	
		Mutant type	Wild-type	Mutant type	Wild-type
Mutant type	17	11	6	13	4
Wild-type	124	3	121	0	124
Sensitivity,% (95% CI)		64.7% (38.3–85.8%)		76.5% (50.1–93.2%)	
Specificity, % (95% CI)		97.6% (93.1–99.5%)		100.0% (97.1–100.0%)	
Accuracy, % (95% CI)		93.6% (88.2–97.0%)		97.2% (92.9–99.2%)	

*NSCLC* non-small cell lung cancer, *TP* true positive, *TN* true negative, *CI* confidence interval

^a^Tissue *EGFR* genotyping results were considered ‘true positive’ or ‘true negative.’ Three cases for which tissue *EGFR* was negative showed positive results using the cobas^®^ *EGFR* Mutation Test v2. These results were regarded as ‘true positive.’

### Effects of storage at cfDNA and short-length exoTNA

**Fig. 3:**
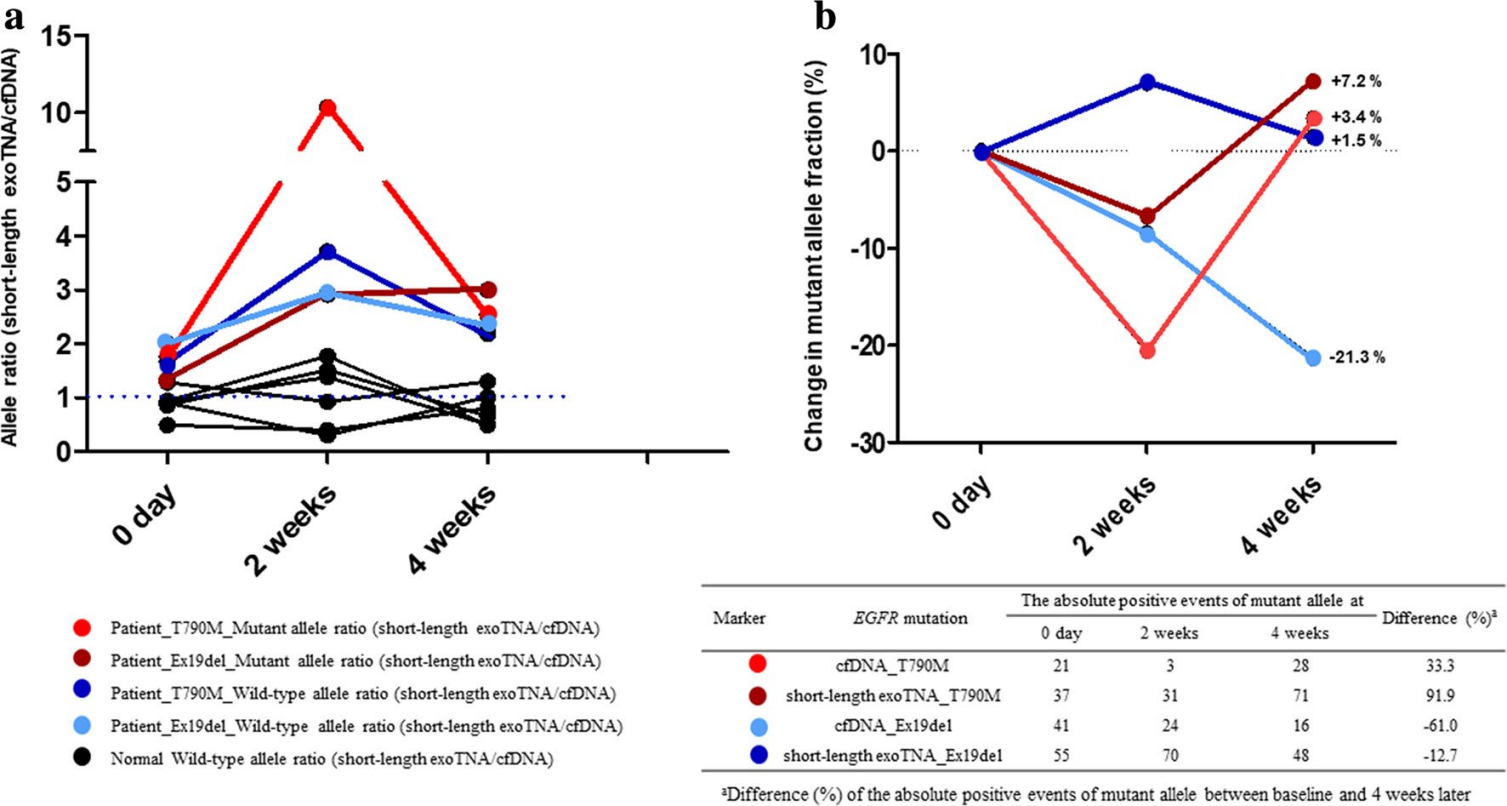
Effects of storage on cfDNA and short-length exoTNA. **a** Allele ratio (short-length exo/cfDNA) change in two patients with *EGFR* mutation and three normal controls. In normal controls, only the wild-type allele ratio (short-length exo/cfDNA) is depicted. **b** The change in mutant allele fraction (%) in short-length exoTNA and cfDNA according to storage time

We determined whether the storage duration of cfDNA and short-length exoTNA could affect the detection of *EGFR* mutations. The median wild-type allele ratio (short-length exoTNA/cfDNA) of three normal controls was 0.9, 1.2 and 0.7 at 0 days, 2 weeks, and 4 weeks, respectively. The average wild-type allele ratio in two patients was 1.8, 3.3, and 2.3 at 0 days, 2 weeks, and 4 weeks, respectively. The average mutant allele ratio was 1.6, 6.6, and 2.8 at 0 days, 2 weeks, and 4 weeks, respectively. The T790M mutant allele ratio showed a fivefold increase after 2 weeks of storage, and this mutant allele ratio at 4 weeks later was similar to that of day 0 (Fig. 3a). The mutant allele copies (Ex19del and T790M) in short-length exoTNA were relatively well preserved after 4 weeks (Fig. 3b). The difference (%) between Ex19del mutant allele fraction (%) and that of the absolute mutant allele copies between 0 days and 4 weeks after storage was − 21.3% and − 61.0%, respectively, in cfDNA (Fig. 3b).

## Discussion

To improve the sensitivity of low levels of plasma cfDNA, there has been a focus on enriching for cfDNA fragments on the basis of length [28–30]. It has been reported that mutant alleles occur more commonly in shorter fragments of cfDNA in lung cancer patients [30]. However, the size of NA in exosomes that might contain relatively high tumor-derived NAs remains unclear. According to Hur et al., *EGFR*-mutant DNA is mainly distributed as high-molecular weight DNA (~ 10 kb) in exosomes from plasma and bronchoalveolar lavage fluid [17]. However, they only evaluated NAs of exosomes using an extraction kit for full-length DNA. Fernando et al. demonstrated that fragmented DNAs of ~ 200 bp in size comprised the majority of NAs localized in exosomes [22]. By comparing different sizes of exoNAs, we observed that the *EGFR* mutant alleles were more enriched in short-length NAs (~ 200 bp long) than in high-molecular weight DNA in exosomes (Additional file 1: Figures S1 and S2).

Recent studies have shown that using single-step isolation of combined exosomal RNA/DNA and cfDNA is a highly sensitive method for detecting *EGFR* mutations in NSCLC patient plasma [16, 18]. However, the target NAs and their size have not been clearly elucidated. We determined that short-length exoTNA is superior to other nucleic acid materials (cfDNA, short-length exoDNA and full-length exoDNA) for mutant allele detection (Table 2 and Fig. 2). When comparing short-length exoTNA to cfDNA, the average number of mutant copies is 1.5 times higher for short-length exoTNA (Fig. 2a). Furthermore, the sensitivity of detecting *EGFR* mutations using short-length exoTNA is higher than that using cfDNA (76.5% vs. 64.7%) (Table 3).

Analysis of cfDNA from blood could be an alternative method for identifying *EGFR* mutations in NSCLC patients. However, detectable mutant copies in cfDNA are too few to increase false negative rate in *EGFR*-mutated NSCLC. Patients with low T790M copy number (< 10 copies/mL) have a similar response to osimertinib to patients with a higher T790M copy number (≥ 10 copies/mL) [31]. Furthermore, an actual number of patients with low T790M copy number (< 10 copies/mL) was fourfold higher than the number of patients with a higher T790M copy number (≥ 10 copies/mL) [32]. Around half of cases in the present study harboring plasma *EGFR* mutation also had low mutant allele copies (< 10 copies/mL) (Fig. 2a, b). In *EGFR*-mutant NSCLC patients with intrathoracic disease (M0/M1a) or cases with low copy T790M, the positive rate was 63.6% (*N* = 7/11) and 45.5% (*N* = 5/11) in short-length exoTNA and cfDNA, respectively (Fig. 2b). These data demonstrate that the increased numbers of detectable *EGFR* mutant copies obtained from extraction of exoTNA could influence the sensitivity of ddPCR-based *EGFR* mutation test for cases with low copy *EGFR* mutants.

Short-length exoTNA and cfDNA showed generally good concordance with tissue *EGFR* results. Despite the small number of studied samples, 17.6% of *EGFR* mutants (*N* = 3/17) were detected in plasma samples where the mutation was not detected in tissue previously. The false-negative results of tissue might be caused by tumor heterogeneity [33] or failure to obtain adequate specimen. This highlights the feasibility of blood-based *EGFR* testing in diagnosis and monitoring of cancer.

To evaluate the influence of storage period on cfDNA and short-length exoTNA for assay performance, we compared cfDNA and exoTNA from plasma at different time points (0, 14, and 28 days). When plasma was stored at − 80 °C for 4 weeks, the amounts of both T790M and Ex19del mutant copies in short-length exoTNA remained stable; however, in cfDNA, Ex19del mutant copies decreased by up to 61%. In the previous study, storage at − 20 °C barely impacted the overall amounts of exosomal miRNAs for at least 5 years [34]. The stability of exoTNA could be explained by the mechanism by which lipid bilayer membrane coating protects internal DNA and RNA [21]. The amounts and integrity of cfDNA could be affected by storage duration. Storing plasma samples at − 80 °C is recommended until further processing of cfDNA isolation is implemented [35]. Following the recommendation, a majority of laboratories have stored plasma at − 80 °C [36]. Barrett et al. demonstrated the stability of cfDNA from plasma samples stored at − 80 °C for up to 2 weeks [35]. However, according to our data, the storage period can influence the stability of cfDNA that is stored more than 2 weeks. Therefore, when performing the *EGFR* assay, the storage duration of plasma should be considered. Given the limited sample size we used, future study should be performed to confirm the effects of − 80 °C storage on the amount and integrity of cfDNA and short-length exoTNA.

## Conclusion

Target nucleic acids and their size distribution might be critical considerations for selecting an extraction method and a detection assay. In this study, we mainly determined the characteristics of an effective target component in exosomes. A shorter exoTNA with 200 bp length contained more detectable tumor-derived nucleic acids than exoDNA (~ 200 bp length or full-length) or cfDNA. Blood-based cancer diagnostic testing is a promising tool not only for early diagnosis of cancer, but also for patient stratification and longitudinal monitoring of residual tumors [5, 37, 38]. Therefore, short-length exoTNA as a sensitive biomarker might be useful to detect *EGFR* mutants for NSCLC patients with low copy numbers of the mutation target.

## Supplementary information

**Additional file 1: Table S1.** Characteristics of the primers and probes as provided by the manufacturer. **Table S2.** The LOD of the ddPCR assay. **Table S3.** Analytical sensitivity of the ddPCR assay. **Figure S1.** Assessment of size-selective target exoNAs related to the sensitivity of *EGFR* mutation testing. **Figure S2.** The distribution of isolated nucleic acids.

## Data Availability

The datasets supporting the conclusions of this article are included within the article and its additional files.

## Abbreviations

NSCLC: non-small cell lung cancer
TKI: tyrosine kinase inhibitor
ctDNA: circulating tumor DNA
exoNA: exosomal nucleic acid
cfDNA: cell-free DNA
exoNA: exosomal nucleic acid
exoTNA: exosomal DNA and RNA
ddPCR: droplet digital polymerase chain reaction
Ex19del: exon 19 deletion
LOD: limitation of detection
CI: confidence interval
WT: wild-type
TP: true positives
TN: true negatives
