# Supplementary material for: Selecting short length nucleic acids localized in exosomes improves plasma EGFR mutation detection in NSCLC patients
Source: Cancer Cell Int. 2019 Oct 1;19:251. doi: 10.1186/s12935-019-0978-8 (PMC6771088; doi:10.1186/s12935-019-0978-8)
Supplement: Supplementary file 1 — Additional file 1: Table S1. Characteristics of the primers and probes as provided by the manufacturer. Table S2. The LOD of the ddPCR assay. Table S3. Analytical sensitivity of the ddPCR assay. Figure S1. Assessment of size-selective target exoNAs related to the sensitivity of EGFR mutation testing. Figure S2. The distribution of isolated nucleic acids. [file 12935_2019_978_MOESM1_ESM.docx]

| **Table S1.** Characteristics of the primers and probes as provided by the manufacturer | | | | | | |
| --- | --- | --- | --- | --- | --- | --- |
| Kit | Chromosome location (hg19) | Amplicon length | COSMIC ID | Nucleotide change of target mutations | Amino acid changes of target mutations | Probe fluorophore |
| PrimePCR™ ddPCR™ Mutation Detection Assay Kit: *EGFR* WT for p.T790M, and *EGFR* p.T790M, Human | chr7:55249042-55249164 | 80 | COSM6240 | c.2369C>T | p.T790M | FAM/HEX |
| PrimePCR™ ddPCR™ Mutation Detection Assay Kit: *EGFR* WT for p.L858R, and *EGFR* p.L858R, Human | chr7:55259483-55259605 | 73 | COSM6224 | c.2573T>G | p.L858R | FAM/HEX |
| PrimePCR™ ddPCR™ *EGFR* Exon 19 Deletions Screening Kit | (-) | (-) | COSM13551, COSM6223, COSM6225, COSM12419, COSM6220, COSM51527, COSM12383, COSM12387, COSM12420, COSM6255, COSM12382, COSM6218, COSM12369 and COSM12370 | 15 deletion mutations in exon 19 (c.2235_2252>AAT, c.2235_2249del15, c.2236_2250del15, c.2238_2252>GCA, c.2238_2255del18, c.2239_2253>CAA, c.2239_2251>C, .2239_2258>CA, c.2239_2252>CA, c.2239_2256del18, c.2239_2248TTAAGAGAAG>C, c.2239_2253del15, c.2239_2247delTTAAGAGAA,  c.2240_2254del15 and c.2240_2257del18) | p.E746_T751>I, p.E746_A750delELREA, p.E746_A750delELREA, p.L747_T751>Q , p.E746_S752>D, p.L747_T751>Q, p.L747_T751>P, .L747_P753>Q, p.L747_T751>Q , p.L747_S752delLREATS , p.L747_A750>P, p.L747_T751delLREAT, p.L747_E749delLRE, p.L747_T751delLREAT and p.L747_P753>S | FAM/HEX |
| Abbreviations: WT, wild-type. | | | | | | |

| **Table S2**. The LOD of the ddPCR assay | | | | | | | | | | | | | | | | | | | | |
| --- | --- | --- | --- | --- | --- | --- | --- | --- | --- | --- | --- | --- | --- | --- | --- | --- | --- | --- | --- | --- |
| Sample # | Blank^a^ | | | | | |  | Healthy control^b^-cfDNA | | | | | |  | Healthy control^b^-exoTNA | | | | | |
|  | L858R | | Exon 19 deletion (ΔE746 - A750) | | T790M | |  | L858R | | Exon 19 deletion (ΔE746 - A750) | | T790M | |  | L858R | | Exon 19 deletion (ΔE746 - A750) | | T790M | |
|  | Wild-type events | Positive type events | Wild-type events | Positive type events | Wild-type events | Positive type events |  | Wild-type events | Positive type events | Wild-type events | Positive type events | Wild-type events | Positive type events |  | Wild-type events | Positive type events | Wild-type events | Positive type events | Wild-type events | Positive type events |
| #1 | 0 | 0 | 0 | 1 | 0 | 0 |  | 68 | 0 | 59 | 0 | 51 | 0 |  | 58 | 0 | 82 | 0 | 44 | 0 |
| #2 | 0 | 0 | 0 | 0 | 0 | 0 |  | 89 | 0 | 87 | 0 | 101 | 0 |  | 100 | 0 | 53 | 0 | 50 | 0 |
| #3 | 0 | 0 | 0 | 0 | 0 | 0 |  | 334 | 0 | 282 | 0 | 172 | 0 |  | 236 | 0 | 252 | 0 | 156 | 0 |
| #4 | 0 | 0 | 0 | 0 | 0 | 0 |  | 69 | 0 | 55 | 0 | 51 | 0 |  | 66 | 0 | 51 | 0 | 44 | 0 |
| #5 | 3 | 0 | 5 | 0 | 2 | 0 |  | 94 | 1 | 86 | 0 | 101 | 0 |  | 84 | 1 | 119 | 1 | 50 | 0 |
| #6 | 0 | 0 | 1 | 0 | 0 | 0 |  | 374 | 0 | 185 | 0 | 172 | 0 |  | 288 | 0 | 185 | 0 | 156 | 0 |
| #7 | 0 | 0 | 0 | 0 | 0 | 0 |  | 85 | 0 | 46 | 0 | 27 | 0 |  | 38 | 0 | 60 | 0 | 41 | 0 |
| #8 | 0 | 0 | 0 | 0 | 0 | 0 |  | 233 | 0 | 373 | 2 | 227 | 0 |  | 233 | 0 | 329 | 0 | 69 | 0 |
| Event |  | 0 |  | 1.0 |  | 0 |  |  | 1.0 |  | 2.0 |  | 0 |  |  | 1.0 |  | 1.0 |  | 0.0 |
| Mean |  | 0 |  | 0.1 |  | 0 |  |  | 0.1 |  | 0.3 |  | 0 |  |  | 0.1 |  | 0.1 |  | 0.0 |
| SD |  | 0 |  | 0.3 |  | 0 |  |  | 0.3 |  | 0.7 |  | 0 |  |  | 0.3 |  | 0.3 |  | 0.0 |
| LOB |  | 0 |  | 1 |  | 0 |  |  |  |  |  |  |  |  |  |  |  |  |  |  |
| LOD (CLSI EP17-A2) | | | | | | |  |  | 0.5 |  | 1.8 |  | 0.0 |  |  | 0.5 |  | 1.2 |  | 0.0 |
| 95% CI upper bound (one-tail Poisson distribution) | | | | | | |  |  | 0.4 |  | 0.6 |  | 0.0 |  |  | 0.4 |  | 0.4 |  | 0.0 |
| ^a^Analysis of blank samples without DNA (n = 8) | | | | | | | | | | | | | | | | | | | | |
| ^b^Eight healthy subjects were anonymized and studied as control samples. | | | | | | | | | | | | | | | | | | | | |
| Abbreviations: LOD, limit of detection; ddPCR, droplet digital polymerase chain reaction; cfDNA, cell-free DNA; exoTNA, exosomal DNA and RNA; SD, standard deviation; LoB, limit of blank. | | | | | | | | | | | | | | | | | | | | |

| **Table S3**. Analytical sensitivity of the ddPCR assay | | | | | | | | | | |
| --- | --- | --- | --- | --- | --- | --- | --- | --- | --- | --- |
| Reference Materials | Variant | Expected allele frequency (%)^a^ | Expected copies of wild-type DNA per sample^a^ | Expected copies of mutant DNA per sample^a^ | 1st ddPCR in house of | | | 2nd ddPCR in house of | | |
|  |  |  |  |  | Allele frequency (%) | Copies of wild-type DNA per sample^b^ | Copies of mutant DNA per sample | Allele frequency (%) | Copies of wild-type DNA per sample^b^ | Copies of mutant DNA per sample |
| 1% Multiplex I cfDNA Reference Standard   (HD778) | L858R | 0.8239 | 3884 | 32 | 1.3225 | 2344 | 31 | 1.0471 | 2483 | 26 |
|  | Ex19del | 1.2259 | 2284 | 28 | 1.3123 | 1524 | 20 | 1.6033 | 1684 | 27 |
|  | T790M | 1.0081 | 1984 | 20 | 1.1500 | 2087 | 24 | 1.1883 | 2188 | 26 |
| 0.1%  Multiplex I cfDNA Reference Standard (HD779) | L858R | 0.0942 | 4248 | 4 | 0.0368 | 2718 | 1 | 0.0631 | 3171 | 2 |
|  | Ex19del | 0.0820 | 2440 | 2 | 0.0538 | 1859 | 1 | 0.1109 | 1803 | 2 |
|  | T790M | 0.1361 | 2204 | 3 | 0.0744 | 2688 | 2 | 0.1581 | 2530 | 4 |
| 0.02%  Multiplex I cfDNA Reference Standard (HD779)^c^ | L858R | 0.0219 | 10942 | 2 | 0.0172 | 11604 | 2 | 0.0152 | 13161 | 2 |
|  | Ex19del | 0.0213 | 9385 | 2 | 0.0299 | 10039 | 3 | 0.0544 | 9195 | 5 |
|  | T790M | 0.0205 | 10242 | 2 | 0.0176 | 11359 | 2 | 0.0092 | 10926 | 1 |
| 0.01%  Multiplex I cfDNA Reference Standard (HD779)^c^ | L858R | 0.0112 | 10099 | 1 | 0.0000 | 11138 | 0 | 0.0000 | 11383 | 0 |
|  | Ex19del | 0.0128 | 8901 | 1 | 0.0325 | 9232 | 3 | 0.0140 | 7120 | 1 |
|  | T790M | 0.0111 | 9805 | 1 | 0.0115 | 8675 | 1 | 0.0297 | 10118 | 3 |
| 0.005%  Multiplex I cfDNA Reference Standard (HD779)^c^ | L858R | 0.0051 | 20215 | 1 | 0.0077 | 12915 | 1 | 0.0130 | 15400 | 2 |
|  | Ex19del | 0.0050 | 17811 | 1 | 0.0080 | 12529 | 1 | 0.0000 | 13877 | 0 |
|  | T790M | 0.0057 | 19619 | 1 | 0.0066 | 15154 | 1 | 0.0073 | 13733 | 1 |
| ^a^Expected mutant allele frequency and copy number values of wild-type and mutant DNA measured using ddPCR were provided by the manufacturer. Expected copy numbers of wild-type DNA in spiked reference materials were calculated with measured copy numbers of fragmented healthy control DNA (~200bp). | | | | | | | | | | |
| ^b^Mean wild-type events of duplicated results at L858R, Ex19del, and T790M were measured using ddPCR. | | | | | | | | | | |
| ^c^cfDNA Reference Standards (Horizon Discovery) with 0.1% mutant alleles were serially diluted to wild-type DNAs. | | | | | | | | | | |
| Abbreviations: ddPCR, droplet digital polymerase chain reaction; Ex19del, exon 19 deletion. | | | | | | | | | | |

**Figure S1.** Assessment of size-selective target exoNAs related to the sensitivity of *EGFR* mutation testing.


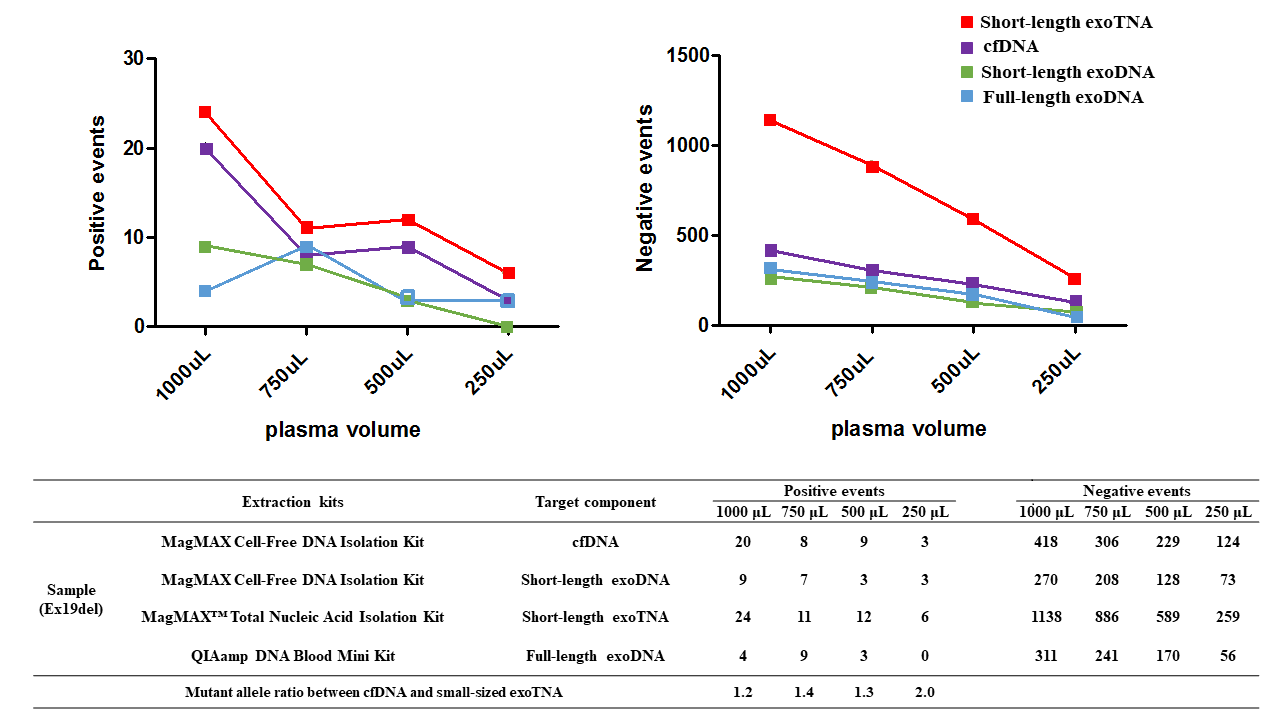


cfDNA, short-length exoNAs (DNA and TNA), and a full-length exoDNA were extracted using different commercial kits. ddPCR was performed with 250, 500, 750, and 1,000 μL plasma samples to assess plasma volume; Ex19del, exon 19 deletion.

**Figure S2.** The distribution of isolated nucleic acids.


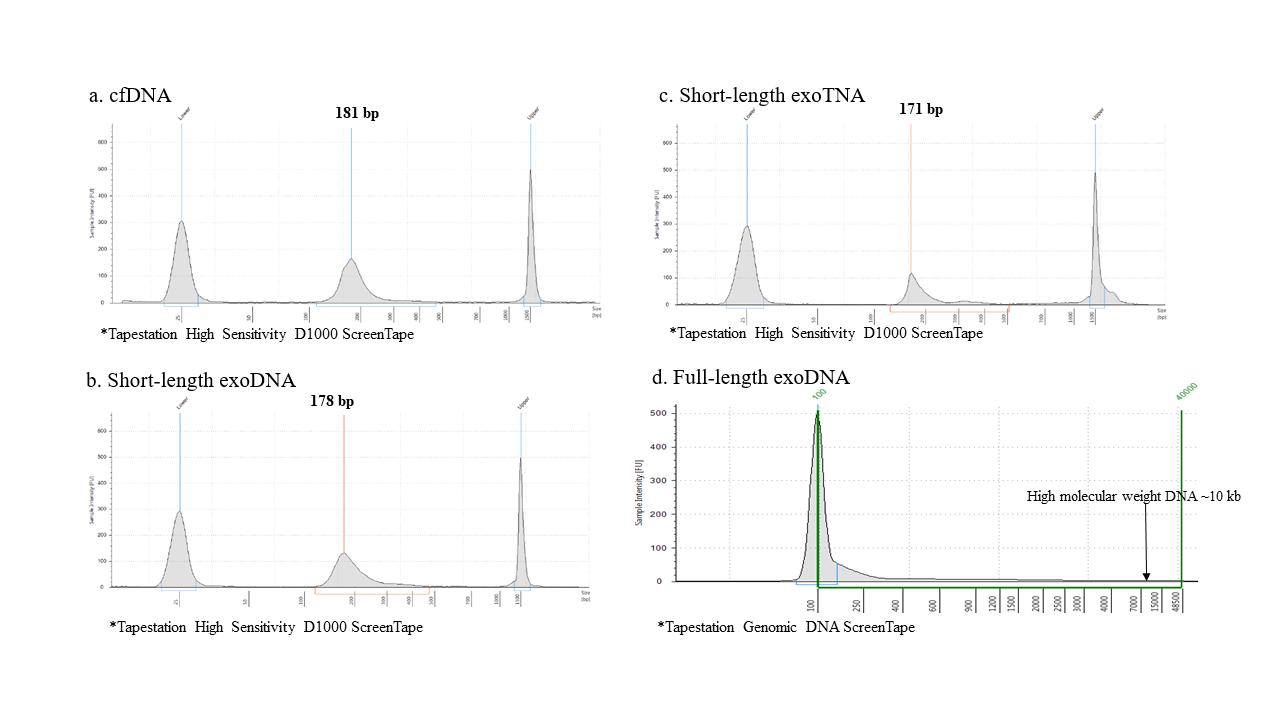


The shorter NAs (~ 200 bp long) were more abundant than high-molecular weight DNA in exosomes. **a.** cfDNA was extracted using a MagMAX Cell-Free DNA Isolation Kit, **b.** short-length exoDNA in isolated exosomes from plasma was extracted using a MagMAX Cell-Free DNA Isolation Kit, **c.** short-length exoTNA in isolated exosomes from plasma was extracted using a MagMAX™ Total Nucleic Acid Isolation Kit, **d.** full-length exoDNA in isolated exosomes from plasma was extracted using a QIAamp DNA Blood Mini Kit.
